# Combined inhibition of bile salt synthesis and intestinal uptake reduces cholestatic liver damage and colonic bile salts in mice

**DOI:** 10.1016/j.jhepr.2023.100917

**Published:** 2023-09-25

**Authors:** Roni F. Kunst, Isabelle Bolt, Rychon D.J. van Dasselaar, Bart A. Nijmeijer, Ulrich Beuers, Ronald P.J. Oude Elferink, Stan F.J. van de Graaf

**Affiliations:** 1 Tytgat Institute for Liver and Intestinal Research, Amsterdam University Medical Centers, University of Amsterdam, Amsterdam, The Netherlands; 2 Amsterdam Gastroenterology, Endocrinology and Metabolism (AGEM), Amsterdam University Medical Centers, Amsterdam, The Netherlands; 3 LinXis BV, Amsterdam, The Netherlands; 4 Department of Gastroenterology and Hepatology, Amsterdam University Medical Centers, University of Amsterdam, Amsterdam, The Netherlands

**Keywords:** ASBT, IBAT, Intestine-restricted ASBT inhibitors, OCA, Cilofexor, NGM282, Fibroblast growth factor 15/19, Cholestasis, Faecal bile salt, Aldafermin, Liver injury, Alkaline phosphatase

## Abstract

**Background & Aims:**

Intestine-restricted inhibitors of the apical sodium-dependent bile acid transporter (ASBT, or ileal bile acid transporter) are approved as treatment for several inheritable forms of cholestasis but are also associated with abdominal complaints and diarrhoea. Furthermore, blocking ASBT as a single therapeutic approach may be less effective in moderate to severe cholestasis. We hypothesised that interventions that lower hepatic bile salt synthesis in addition to intestinal bile salt uptake inhibition provide added therapeutic benefit in the treatment of cholestatic disorders. Here, we test combination therapies of intestinal ASBT inhibition together with obeticholic acid (OCA), cilofexor, and the non-tumorigenic fibroblast growth factor 15 (Fgf15)/fibroblast growth factor 19 (FGF19) analogue aldafermin in a mouse model of cholestasis.

**Methods:**

Wild-type male C57Bl6J/OlaHsd mice were fed a 0.05% 3,5-diethoxycarbonyl-1,4-dihydrocollidine (DDC) diet and received daily oral gavage with 10 mg/kg OCA, 30 mg/kg cilofexor, 10 mg/kg ASBT inhibitor (Linerixibat; ASBTi), or a combination. Alternatively, wild-type male C57Bl6J/OlaHsd mice were injected with adeno-associated virus vector serotype 8 (AAV8) to express aldafermin, to repress bile salt synthesis, or to control AAV8. During a 3-week 0.05% DDC diet, mice received daily oral gavage with 10 mg/kg ASBTi or placebo control.

**Results:**

Combination therapy of OCA, cilofexor, or aldafermin with ASBTi effectively reduced faecal bile salt excretion. Compared with ASBTi monotherapy, aldafermin + ASBTi further lowered plasma bile salt levels. Cilofexor + ASBTi and aldafermin + ASBTi treatment reduced plasma alanine transaminase and aspartate transaminase levels and fibrotic liver immunohistochemistry stainings. The reduction in inflammation and fibrogenesis in mice treated with cilofexor + ASBTi or aldafermin + ASBTi was confirmed by gene expression analysis.

**Conclusions:**

Combining pharmacological intestinal bile salt uptake inhibition with repression of bile salt synthesis may form an effective treatment strategy to reduce liver injury while dampening the ASBTi-induced colonic bile salt load.

**Impact and Implications:**

Combined treatment of intestinal ASBT inhibition with repression of bile salt synthesis by farnesoid X receptor agonism (using either obeticholic acid or cilofexor) or by expression of aldafermin ameliorates liver damage in cholestatic mice. In addition, compared with ASBT inhibitor monotherapy, combination treatments lower colonic bile salt load.

## Highlights

• Combinations of FXR agonism and intestinal ASBT inhibition ameliorate cholestasis-induced liver injury in mice.
• ASBT inhibition with AAV-mediated aldafermin treatment lowered liver damage, inflammation, and fibrosis markers.
• Combination treatments lower colonic bile salt load, which may reduce the risk of diarrhoea.

## Graphical abstract

**Figure:**
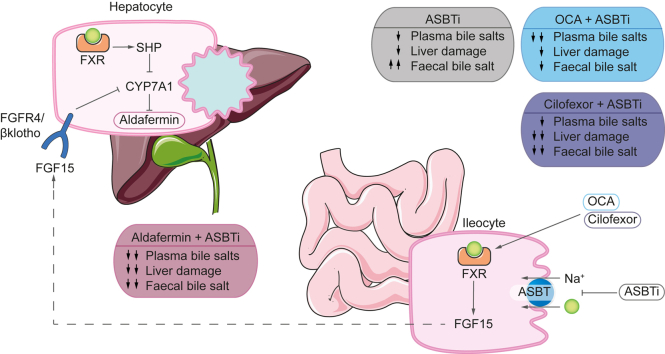

## Introduction

Cholestasis is a critical condition in which bile flow from the liver towards the small intestine is disturbed or completely blocked owing to a variety of underlying diseases. Cholestasis is observed in people diagnosed with primary biliary cholangitis (PBC)^1^ or primary sclerosing cholangitis (PSC),^2^ where inflammation of the bile ducts plays a role. Bile flow is also impaired in individuals with progressive familial intrahepatic cholestasis (PFIC)^3^ or Alagille syndrome,^4^ caused by congenital defects in transporters or bile duct anatomy. If cholestasis remains untreated, toxic accumulation of bile acids in the liver can lead to severe liver damage that may ultimately require liver transplantation to prevent death as a result of terminal liver failure.^3^

Current treatments focus on dampening liver injury, thereby not only preventing end-stage liver disease, but also ameliorating symptoms for patients such as cholestasis-associated itch. Steroidal and non-steroidal farnesoid X receptor (FXR) agonists such as the bile salt-based obeticholic acid (OCA) and cilofexor, which has a non-steroidal structure, induce fibroblast growth factor 15 (Fgf15)/fibroblast growth factor 19 (FGF19) signalling to the liver, repressing cholesterol 7 alpha-hydroxylase (CYP7A1), the rate-limiting enzyme for *de novo* bile salt synthesis. Combined with ursodeoxycholic acid (UDCA), 12-month cotreatment with OCA reduces the prognostic markers alkaline phosphatase and total bilirubin levels in serum of people with PBC.^5^ More recently, aldafermin, a non-tumorigenic FGF19 analogue,^6^ lowered the concentration of toxic hydrophobic serum bile salts and improved markers of fibrosis in a phase II trial in PSC.^7^ Cilofexor also improved markers of liver injury in a phase II clinical trial in people with PSC.^8^ However, an interim analysis of the phase III PRIMIS study testing cilofexor in patients with PSC showed low probability (≤10%) of achieving its primary endpoint of a reduced risk of fibrosis progression, leading to discontinuation of the study.^9^

Other treatment strategies focus on interrupting the enterohepatic circulation.[10], [11], [12] The apical sodium-dependent bile salt transporter (ASBT, also known as the ileal bile acid transporter [IBAT]) is highly expressed in the small intestine, where it takes up bile salts back into the ileocyte, after which bile salts are transported via the portal vein back to the liver. Clinical trials in children with congenital cholestatic syndromes have shown that pruritus as a result of cholestasis is reduced by using ASBT inhibitors (ASBTi) and recently, two different intestine-restricted ASBTi, maralixibat^13^ and odevixibat,^14^ have been approved by the FDA and EMA for treatment of Alagille syndrome and PFIC, respectively.^15^^,^^16^

Unfortunately, most of the treatments described above are also associated with adverse events, such as (worsening of cholestasis-associated) itch with OCA treatment, hypercholesterolaemia with OCA and aldafermin in individuals with non-alcoholic steatohepatitis, and diarrhoea with ASBT inhibition. The latter is likely caused by the high bile salt load in the colon.^17^ Furthermore, inhibiting ASBT in severe cases of cholestasis might not be effective as low amounts of bile salts reach the terminal ileum for re-uptake in that condition.^16^

For that reason, we previously postulated that compared with monotherapy, a combination of OCA and ASBTi might be beneficial for patients with PSC owing to increased efficacy and a lower risk for developing unwanted side effects.^18^ In the present study, we test this experimentally by combining ASBTi in a preclinical cholestasis model with three clinically relevant treatment strategies to reduce bile salt synthesis, to reduce liver injury, and to lower the elevated colonic bile salt load observed during ASBTi monotherapy. We investigated both OCA and the nonsteroidal cilofexor as pharmacological FXR agonists and expressed aldafermin by adeno-associated virus (AAV) to target signalling pathways downstream of FXR.

## Materials and methods

### Animals and experimental design

Adult wild-type male mice (C57BL/6JOlaHSD) were purchased from Envigo and were fed C1000 control diet (Altromin, Lage, Germany) enriched with 0.01% 3,5-diethoxycarbonyl-1,4-dihydrocollidine (DDC; Sigma, Houten, The Netherlands; and Triple A Trading, Tiel, The Netherlands) for 1 week, followed by 2 weeks C1000 control diet enriched with 0.05% DDC (Triple A Trading). Both diets contain 80 g/kg maltodextrin and 110 g/kg cream to increase palatability. During the DDC diet, mice were treated daily with a combination of 10 mg/kg OCA^19^^,^^20^ (INT-747, MedChemExpress), 30 mg/kg cilofexor^21^ (GS-9674, MedChemExpress), or placebo at 8 a.m. and 10 mg/kg intestinal ASBTi (Linerixibat; MedChemExpress) or placebo by oral gavage at 5 p.m. Placebo treatment consisted of 1% methylcellulose (8 a.m.) or 0.5% hydroxypropyl methylcellulose, 0.1% Tween-80 (Applichem, Darmstadt, Germany) (5 p.m.). At the end of the experiment, mice were treated with placebo/OCA/cilofexor before starting a 4-h fast. Next, mice were anaesthetised as described before, and blood and organs were collected.^19^ In a second experiment, adult wild-type male mice (C57BL/6JOlaHSD, Envigo) were administered either NGM282/aldafermin-encoding^6^^,^^11^ AAV or pTRCGW (control) in a dosage of 1 × 10^11^ genome copies/mouse. Three weeks after injecting AAV, mice were fed a 0.05% DDC diet for 19 days while receiving daily oral gavage with ASBTi or placebo control (0.5% hydroxypropyl methylcellulose, 0.1% Tween-80) at 5 p.m. Mice were sacrificed as described before.^19^ During the experiment, mice were co-housed with two to five mice per cage. Mice were individually housed for 24 h at the final day of the experiment. All mice were kept on a 12-h light/12-h dark cycle with *ad libitum* access to food and water, and experiments have been performed at the Academic Medical Center. The study design and animal care and handling were approved by the Institutional Animal Care and Use Committee of the University of Amsterdam.

### Statistics

Data are provided as mean ± SD. Data points represent individual mice. Differences between groups are calculated using a Kruskal–Wallis one-way ANOVA in the case of four or more groups. Here, we test (i) whether treatment is effective in reducing cholestasis-induced liver injury and (ii) whether combined treatment is more effective than monotherapy. Effectively, this results in six comparisons for the OCA/cilofexor study (placebo *vs*. OCA + ASBTi; placebo *vs*. cilofexor + ASBTi; OCA *vs*. OCA + ASBTi; cilofexor *vs*. cilofexor + ASBTi; placebo *vs*. OCA; and placebo *vs.* cilofexor) and four comparisons for the aldafermin study (placebo *vs.* aldafermin; placebo *vs*. aldafermin + ASBTi; ASBTi *vs*. aldafermin + ASBTi; and aldafermin *vs*. aldafermin + ASBTi). When there are only two treatment groups, the non-parametric Mann–Whitney *U* test was performed. A *p* value of ≤0.05 is considered statistically significant and is calculated using GraphPad Prism v9.0 (GraphPad Software Inc., San Diego, CA, USA).

Further details can be found in the Supplementary Materials and methods and the Supplementary CTAT Table.

## Results

### Improved liver enzymes for liver injury with cilofexor monotherapy and cilofexor + ASBTi combination therapy

**Fig. 1:**
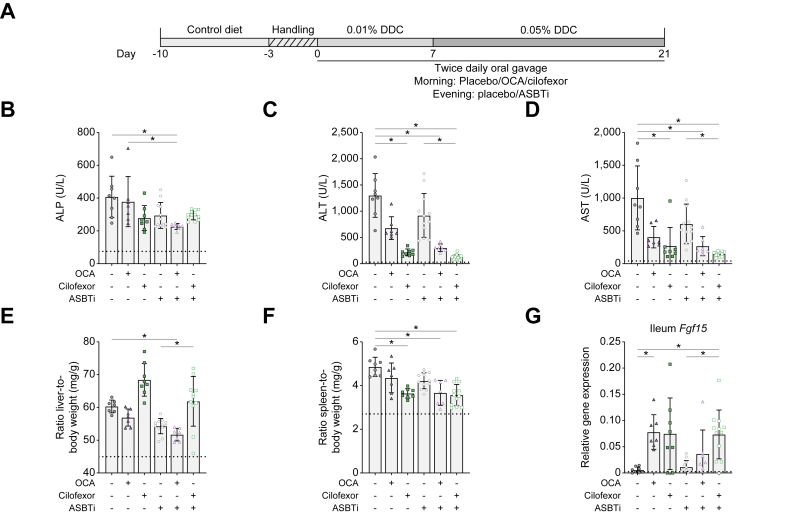
OCA + ASBTi and mostly cilofexor + ASBTi reduce plasma liver injury markers. (A) Schematic overview of the experiment. (B) Plasma ALP. (C) Plasma ALT. (D) Plasma AST. (E) Liver weight. (F) Spleen weight. (G) Ileum *Fgf15* gene expression relative to *Gapdh*. Data are shown as mean ± SD, and individual data points represent individual mice. Healthy controls (n = 3) are indicated by the dotted line. Statistical differences were measured using a Kruskal–Wallis one-way ANOVA, ∗*p* ≤0.05. ALP, alkaline phosphatase; ALT, alanine transaminase; ASBT, apical sodium-dependent bile acid transporter; ASBTi, ASBT inhibitor; AST, aspartate transaminase; DDC, 3,5-diethoxycarbonyl-1,4-dihydrocollidine; *Fgf15*, fibroblast growth factor 15; *Gapdh*, glyceraldehyde 3-phosphate dehydrogenase; OCA, obeticholic acid.

To prevent acute aversive reduction in food intake as a result of the DDC diet, mice were first placed on chow with similar consistency and handled similarly as during the intervention period. Subsequently, mice received a run-in concentration of 0.01% DDC diet in the first week (Fig. 1A). Total bodyweight of the mice was not affected by the 0.01% DDC diet in Week 1, and the 0.05% DDC diet caused a gradual ±5–10% bodyweight loss in 4 days, after which their body weight stabilised (Fig. S1A). In vehicle-treated conditions, liver enzymes alkaline phosphatase (ALP), alanine transaminase (ALT), and aspartate transaminase (AST) reached highly elevated levels, and liver-to-body weight ratio and spleen-to-body weight ratio were increased, thus confirming the cholestatic phenotype in this model (Fig. 1B–F). Intestinal FXR activation was confirmed by the *Fgf15* gene expression in the ileum, which was induced in the OCA- or cilofexor-treated groups (Fig. 1G).

Compared with OCA monotherapy and placebo control, OCA + ASBTi lowered plasma ALP levels, but compared with ASBTi monotherapy, OCA + ASBTi did not change plasma ALP levels. Furthermore, OCA + ASBTi treatment lowered ALT and AST compared with placebo (*p* = 0.0212 and *p* = 0.0327, respectively). Compared with the ASBTi monotherapy group, ALT and AST were numerically lowered by 67 and 56%, respectively, by OCA + ASBTi combination therapy. However, this did not reach significance. Compared with placebo treatment, cilofexor monotherapy or combination treatment with ASBTi did not affect plasma ALP levels but reduced both ALT and AST levels. In addition, compared with mice that received ASBTi monotherapy, mice treated with cilofexor + ASBTi showed improved ALT and AST, suggesting improved liver health. Furthermore, ALT levels were numerically reduced with 40% (group average from 214 to 128 U/L) and AST levels with 45% (from 271 to 147 U/L) in mice treated with cilofexor + ASBTi compared with mice that received cilofexor monotherapy, suggesting added benefit of the combination. However, this was not a statistically significant difference.

Compared with placebo-treated mice, OCA + ASBTi-treated mice had decreased liver weight, but compared with ASBTi monotherapy, the cilofexor + ASBTi combination increased liver weight (Fig. 1E). Spleen weight was reduced because of cilofexor, OCA + ASBTi, and cilofexor + ASBTi treatments compared with placebo, suggesting that there is reduced inflammation in the aforementioned treatment groups (Fig. 1F).

As conjugated OCA is an ASBT substrate, this may lead to reduced exposure.^19^ However, compared with placebo, OCA also showed increased *Fgf15* expression, implying that 10 mg/kg/day OCA activates intestinal FXR (Fig. 1G). Note that 6 of 12 mice in the first cohort first received two dosages of 30 mg/kg/day, but owing to >15% bodyweight loss in combination with discomfort-revealing behaviour in the other six mice, it was necessary to lower the dosage to 10 mg/kg/day for the remainder of the experiment (Fig. S1B and Supplementary Materials and methods). The post-mortem section of early-sacrificed mice receiving 30 mg/kg/day OCA showed bloating of the gastrointestinal tract (Fig. S1C).

### Minor fibrosis after 3-week DDC diet and reduced inflammation caused by cilofexor and cilofexor + ASBTi combination treatment

**Fig. 2:**
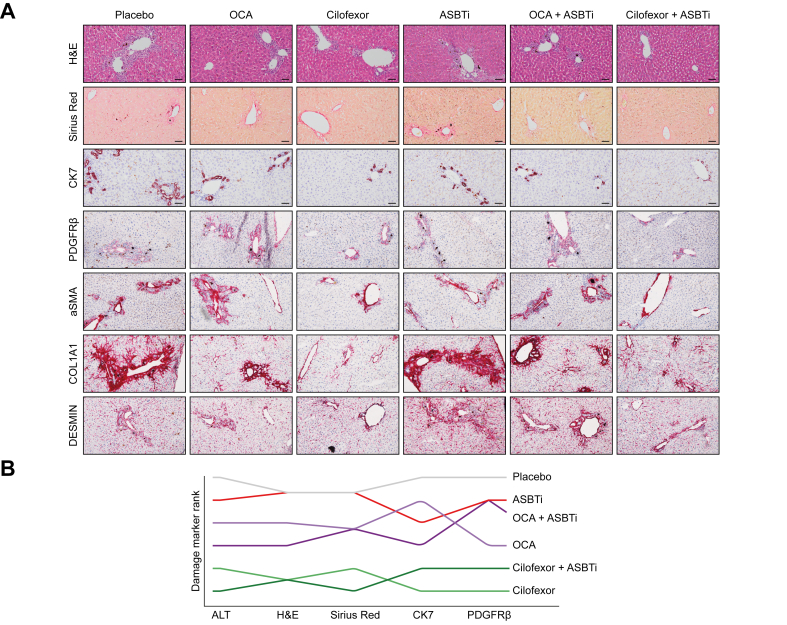
Improved liver health with cilofexor and cilofexor + ASBTi treatment. (A) H&E, Sirius Red, CK7, PDGFRβ, αSMA, COL1A1, and DESMIN stain of liver tissue; the scale bar represents 50 μm. (B) Ranking of liver injury by individual treatments. αSMA, alpha smooth muscle actin; ASBT, apical sodium-dependent bile acid transporter; ASBTi, ASBT inhibitor; CK7, cytokeratin 7; COL1A1, collagen type 1 alpha 1; OCA, obeticholic acid; PDGFRβ, platelet-derived growth factor receptor beta.

To further assess cholestasis-induced liver injury, histological stainings were performed on liver tissue (Fig. 2A). Ductular reaction was most pronounced in the placebo- and ASBTi-treated groups, as evident from the immune cell infiltration in the portal area surrounding bile ducts. OCA and cilofexor monotherapy as well as OCA + ASBTi combination treatment showed less ductular reaction; however, cilofexor + ASBTi combination treatment showed the least immune cell infiltration. Fibrosis, as visualised by Sirius Red staining, was relatively mild in all treatment groups; nevertheless, it was clear that cilofexor monotherapy and cilofexor + ASBTi combination therapy lowered fibrosis compared with placebo-treated mice and ASBTi monotherapy. Cytokeratin 7 (CK7), platelet-derived growth factor receptor beta (PDGFRβ), alpha smooth muscle actin (αSMA), collagen type 1 alpha 1 (COL1A1), and DESMIN-positive stain were also markedly reduced because of cilofexor and cilofexor + ASBTi treatments. A ranking of the intervention groups based on various liver injury markers including plasma ALT and histological stainings is displayed in Fig. 2B. The stainings were assessed in a blinded manner, by at least two people.

**Fig. 3:**
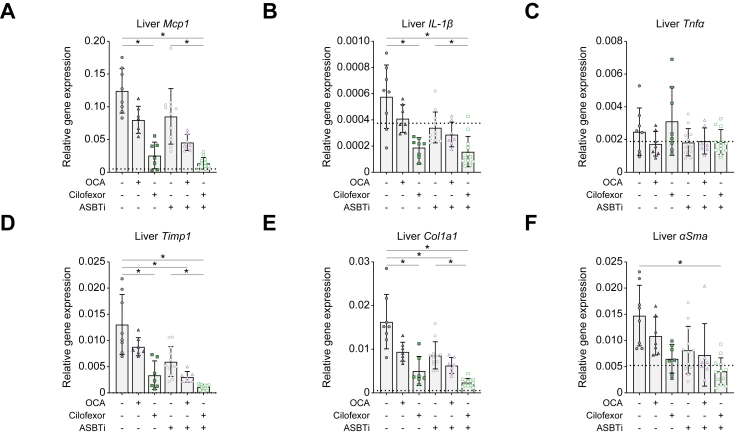
Cilofexor + ASBTi combination treatment improves liver inflammatory and fibrotic markers. compared with ASBTi monotherapy. Liver mRNA expression of (A) *Mcp1*, (B) *Il-1β,* (C) *Tnfα*, (D) *Timp1*, (E) *Col1a1*, and (F) *αSma,* relative to *Gapdh*. Data are shown as mean ± SD, and individual data points represent individual mice. Healthy controls (n = 3) are indicated by the dotted line. Statistical differences were measured using a Kruskal–Wallis one-way ANOVA, ∗*p* ≤0.05. *αSma*, alpha smooth muscle actin; ASBT, apical sodium-dependent bile acid transporter; ASBTi, ASBT inhibitor; *Col1a1*, collagen type 1 alpha 1; *Gapdh*, glyceraldehyde 3-phosphate dehydrogenase; *Mcp1*, monocyte chemoattractant protein-1; OCA, obeticholic acid; *Timp1*, TIMP metallopeptidase inhibitor 1; *Tnfα*, tumour necrosis factor alpha.

Hepatic gene expression of the inflammatory markers monocyte chemoattractant protein-1 (*Mcp1*) and *Il-1β* was decreased because of cilofexor + ASBTi combination therapy compared with both placebo and ASBTi monotherapy, although cilofexor treatment alone was almost equally effective (Fig. 3A and B). Tumour necrosis factor alpha (*Tnfα*) levels did not differ between groups (Fig. 3C).

Gene expression of fibrosis markers TIMP metallopeptidase inhibitor 1 (*Timp1*) and *Col1a1* was decreased in the liver because of cilofexor + ASBTi combination therapy compared with both placebo and ASBTi monotherapy (Fig. 3D and E). *αSma* was lower in the cilofexor + ASBTi combination therapy group than in the placebo treatment group (Fig. 3F). Compared with vehicle only, OCA + ASBTi combination treatment effectively reduced *Timp1* and *Col1a1*, but no additive effect was observed in the OCA + ASBTi combination treatment group compared with the single (OCA/ASBTi) treatment groups. Single or combination treatment with OCA did not affect expression of other inflammatory or fibrosis-related marker genes (Fig. 3A and B).

### Reduced faecal bile salt excretion by cilofexor + ASBTi combination therapy compared with ASBTi alone

**Fig. 4:**
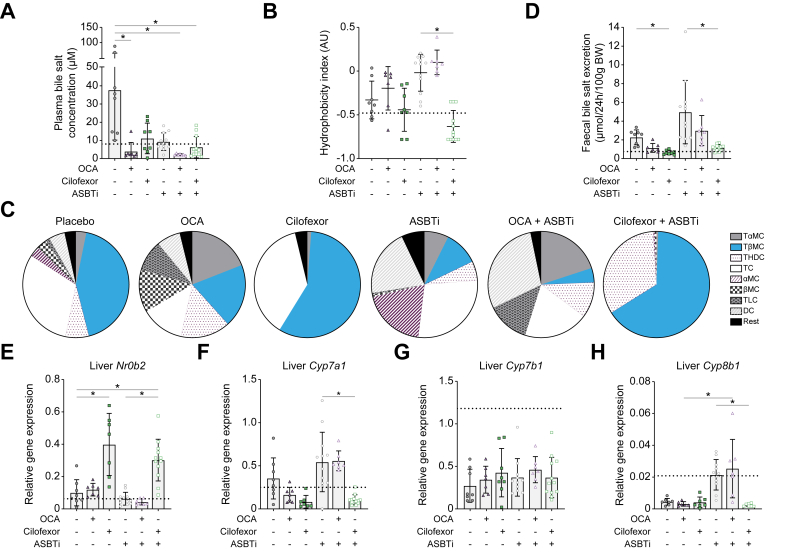
Cilofexor + ASBTi lowered plasma bile salt concentration and hydrophobicity and repressed faecal bile salt excretion. (A) Plasma bile salt concentration. (B) Plasma hydrophobicity index. (C) Plasma bile salt composition. (D) Faecal bile salt excretion. mRNA expression of liver (E) *Shp*, (F) *Cyp7a1*, (G) *Cyp7b1*, and (H) *Cyp8b1* relative to *Gapdh*. Data are shown as mean ± SD, and individual data points represent individual mice. Healthy controls (n = 3) are indicated by the dotted line. Statistical differences were measured using a Kruskal–Wallis one-way ANOVA, ∗*p* ≤0.05. αMC, α-muricholic acid; ASBT, apical sodium-dependent bile acid transporter; ASBTi, ASBT inhibitor; βMC, β-muricholic acid; BW, body weight; *Cyp7a1*, cholesterol 7 alpha-hydroxylase; *Cyp7b1*, cholesterol 7 beta-hydroxylase; *Cyp8b1*, sterol 12-alpha-hydroxylase; DC, deoxycholic acid; *Gapdh*, glyceraldehyde 3-phosphate dehydrogenase; OCA, obeticholic acid; *Shp*, small heterodimer particle (or *Nr0b2*); TɑMC, taurine-conjugated α-muricholic acid; TβMC, taurine-conjugated β-muricholic acid; TC, taurine-conjugated cholic acid; THDC, taurine-conjugated hyodeoxycholic acid; TLC, taurine-conjugated lithocholic acid.

Plasma bile salt concentration in placebo-treated mice was four to five times higher than that in healthy controls (Fig. 4A). Compared with placebo treatment, OCA, OCA + ASBTi, and cilofexor + ASBTi treatments reduced plasma bile salt concentrations, and in the case of OCA and OCA + ASBTi, values were even below those of healthy controls. The composition of circulating bile salts was measured to assess whether FXR-mediated repression of bile salt synthesis affected bile pool hydrophobicity. Only cilofexor + ASBTi lowered the hydrophobicity index, implying that mice treated with cilofexor + ASBTi maintain a more hydrophilic and therefore less toxic plasma bile salt composition (Fig. 4B). The plasma bile salt pool of mice treated with cilofexor + ASBTi mostly comprised taurine-conjugated β-muricholic acid and taurine-conjugated hyodeoxycholic acid, whereas the plasma bile salt composition of placebo- or ASBTi-treated mice was much more diverse (Fig. 4C). Blocking bile salt resorption with ASBTi results in ∼2.5-fold increased faecal bile salt excretion in ASBTi-treated mice compared with placebo-treated mice (Fig. 4D). Compared with ASBTi monotherapy, only cilofexor + ASBTi combination therapy lowered faecal bile salt concentration (*p* = 0.0007).

The difference in bile salt composition between the OCA and cilofexor groups may be related to differences in hepatic FXR activation, visualised in expression levels of small heterodimer particle (*Shp*, or *Nr0b2*). Compared with vehicle treatment, OCA treatment (alone or in combination with ASBTi) did not induce *Shp*, in contrast to cilofexor alone and cilofexor + ASBTi combination therapy (Fig. 4E). Compared with ASBTi monotherapy, cilofexor + ASBTi combination therapy repressed liver *Cyp7a1* (Fig. 4F). The effects of OCA on *Cyp7a1* and *Shp* expression were less clear, particularly in combination with ASBTi. None of the treatment groups restored *Cyp7b1* expression levels (Fig. 4G). Compared with OCA monotherapy, OCA + ASBTi treatment displayed elevated sterol 12-alpha-hydroxylase (*Cyp8b1*) expression, but cilofexor dampened the hepatic *Cyp8b1* induction observed upon ASBT inhibition (Fig. 4H).

Transcripts encoding the organic solute transporter beta (OSTβ*;* gene solute carrier family 51 subunit beta [*Slc51b*]) and bile salt export pump (BSEP; gene ATP binding cassette subfamily B member 11 [*Abcb11*]) as well as mRNA encoding the Na^+^-taurocholate cotransporting polypeptide (NTCP) (gene: solute carrier family 10 member 1 [*Slc10a1*]) in the liver were increased compared with vehicle when mice received cilofexor + ASBTi combination treatment (Fig. S2A–C). In general, expression of *Slc10a1* was low compared with that in healthy controls, most likely an FXR-induced hepatoprotective effect as a result of cholestasis. Gene expression of multidrug resistance-associated protein 2 (MRP2; gene ATP-binding cassette sub-family C member 2 [*Abcc2*]) was not changed because of treatment (Fig. S2D). BSEP protein levels were not affected by treatment, whereas protein expression of NTCP was increased because of cilofexor + ASBTi treatment compared with vehicle (Fig. S2E). OCA + ASBTi also tended to partially restore NTCP protein levels, although this increase was not significant compared with vehicle (*p* = 0.055). In the ileum, organic solute transporter alpha (OSTα; gene solute carrier family 51 subunit alpha [*Slc51a*]) and *Slc51b* were increased in mice treated with cilofexor + ASBTi compared with mice that received ASBTi monotherapy, whereas expression of the ileal bile acid binding protein (IBABP; gene fatty acid binding protein 6 [*Fabp6*]) and IBAT (ASBT; gene solute carrier family 10 member 2 [*Slc10a2*]) was not affected by any treatment (Fig. S2F–I).

### Combination therapy aldafermin + ASBTi improves plasma liver enzymes and inflammatory gene expression in the liver during cholestasis

**Fig. 5:**
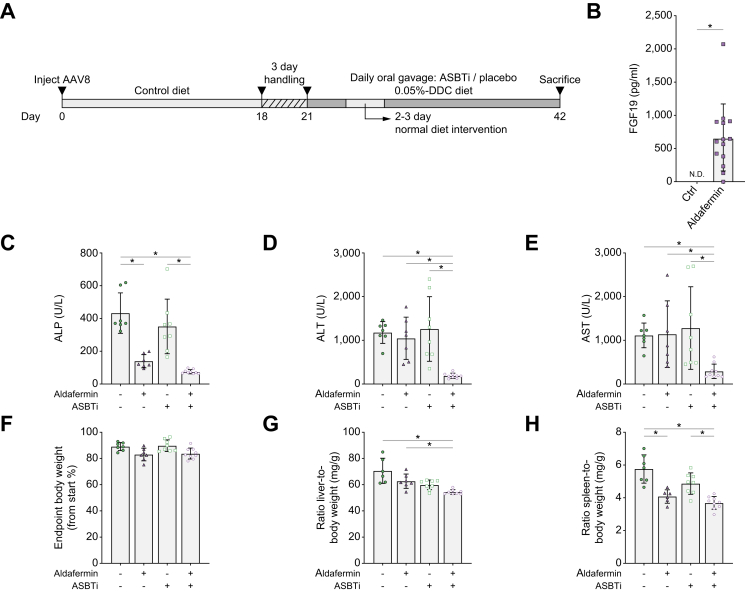
Aldafermin AAV elevated plasma FGF19 and improves cholestatic markers. (A) Schematic overview of the experiment. (B) Plasma FGF19 3 weeks after AAV8 injection. Statistical differences were measured using a non-parametric Mann–Whitney *U* test, ∗*p* ≤0.05. (C) Plasma ALP. (D) Plasma ALT. (E) Plasma AST. (F) Endpoint bodyweight. (G) Liver weight. (H) Spleen weight. Data are shown as mean ± SD, and individual data points represent individual mice. Statistical differences were measured using a Kruskal–Wallis one-way ANOVA, ∗*p* ≤0.05. AAV, adeno-associated virus; AAV8, AAV vector serotype 8; ALP, alkaline phosphatase; ALT, alanine transaminase; ASBT, apical sodium-dependent bile acid transporter; ASBTi, ASBT inhibitor; AST, aspartate transaminase; DDC, 3,5-diethoxycarbonyl-1,4-dihydrocollidine; FGF19, fibroblast growth factor 19.

In a second strategy, ASBT inhibition was combined with pharmacological inhibition of bile salt synthesis in a manner independent of FXR agonism, using aldafermin, previously known as NGM282 (Fig. 5A). Aldafermin is a non-tumorigenic FGF19 analogue, and its expression was confirmed by an FGF19 ELISA 3 weeks subsequent to aldafermin AAV vector serotype 8 (AAV8) administration (Fig. 5B). Induction of cholestasis as a result of the DDC diet was confirmed by a strong increase in plasma liver injury markers ALP, ALT, and AST (Fig. 5C–E). ALP levels were decreased in both the aldafermin and aldafermin + ASBTi groups (Fig. 5C). ALT and AST levels were only decreased upon combination treatment with aldafermin + ASBTi (Fig. 5D and E). A modest reduction in bodyweight was seen in all groups (Fig. 5F), where the increase in liver size as a result of the DDC diet was dampened in the aldafermin + ASBTi group compared with both the vehicle and ASBTi monotherapy groups (Fig. 5G). A reduced spleen size, suggesting less inflammation, was seen in the aldafermin monotherapy group as well as upon aldafermin + ASBTi combination therapy (Fig. 5H).

**Fig. 6:**
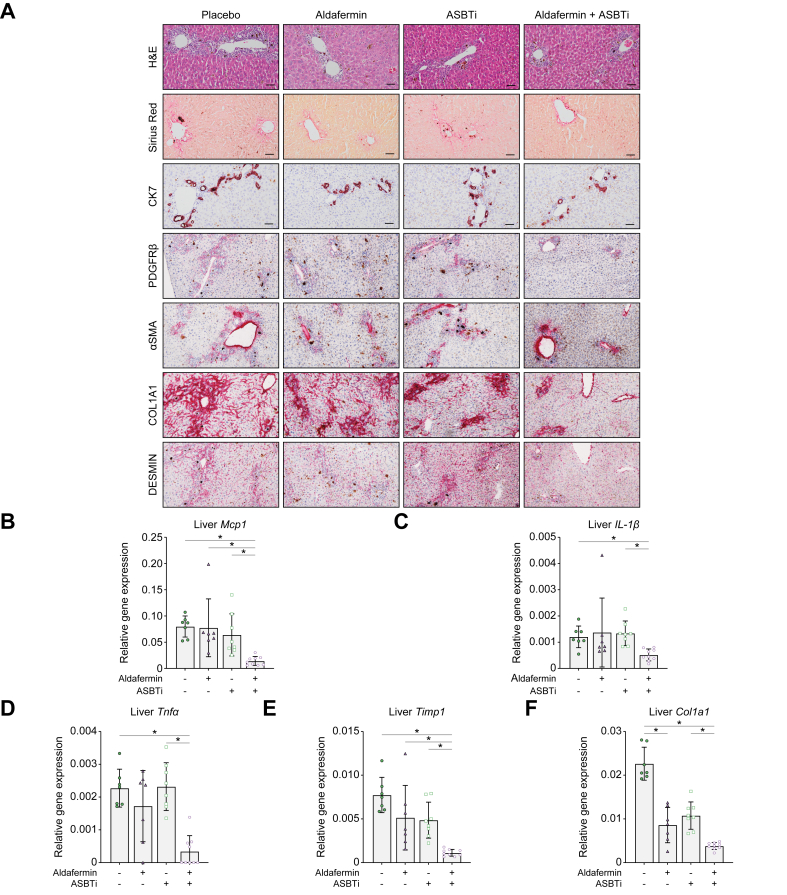
Aldafermin ameliorates cholestasis-induced liver injury only in combination with ASBTi. (A) H&E, Sirius Red, CK7, PDGFRβ, αSMA, COL1A1, and DESMIN stain of liver tissue; the scale bar represents 50 μm. Liver mRNA expression of (B) *Mcp1*, (C) *Il-1β*, (D) *Tnfα*, (E) *Timp1*, and (F) *Col1a1* relative to *Gapdh*. Data are shown as mean ± SD, and individual data points represent individual mice. Statistical differences were measured using a Kruskal–Wallis one-way ANOVA, ∗*p* ≤0.05. αSMA, alpha smooth muscle actin; ASBT, apical sodium-dependent bile acid transporter; ASBTi, ASBT inhibitor; CK7, cytokeratin 7; COL1A1, collagen type 1 alpha 1; *Gapdh*, glyceraldehyde 3-phosphate dehydrogenase; *Mcp1*, monocyte chemoattractant protein-1; *Timp1*, TIMP metallopeptidase inhibitor 1; *Tnfα*, tumour necrosis factor alpha; PDGFRβ, platelet-derived growth factor receptor beta.

All groups showed mild ductular reaction in the periportal regions, but this was markedly reduced because of aldafermin AAV monotherapy (Fig. 6A). Fibrosis, visualised by Sirius Red staining, was mild and not different because of treatment, whereas CK7-positive stain was reduced in the aldafermin monotherapy- and combination-treated groups compared with placebo control. These observations were similar for PDGFRβ, αSMA, COL1A1, and DESMIN-positive stain.

Furthermore, combination treatment aldafermin + ASBTi decreased mRNA expression levels of inflammatory markers *Mcp1*, *Il-1β*, and *Tnfα* and fibrotic markers *Timp1* and *Col1a1* compared with placebo and to ASBTi monotherapy (Fig. 6B–F). Taken together, this suggests that aldafermin + ASBTi combination treatment is more effective in improving liver health than ASBTi monotherapy.

### Repressed bile salt synthesis, transport, and signalling as a result of aldafermin treatment

**Fig. 7:**
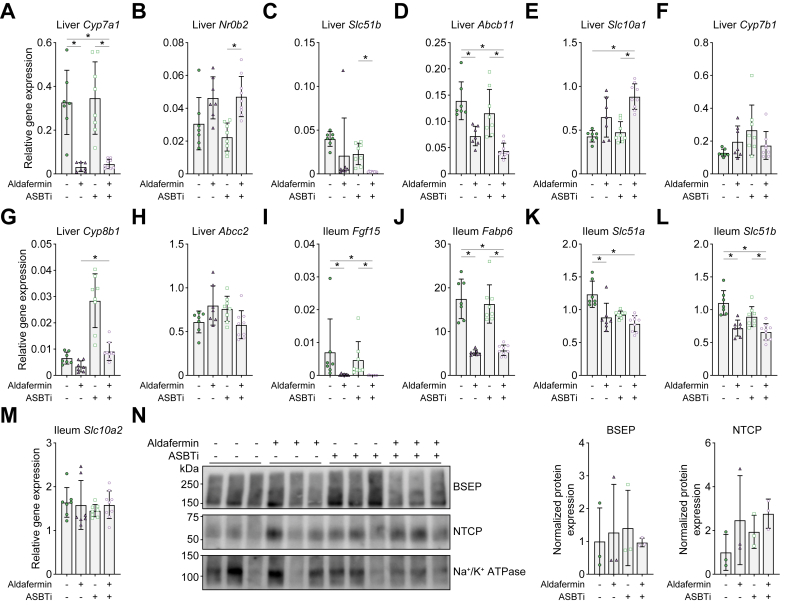
Aldafermin repressed not only bile salt synthesis but also bile salt transport and signalling. Gene expression of (A) liver *Cyp7a1*, (B) liver *Shp*, (C) liver *Slc51b*, (D) liver *Abcb11*, (E) liver *Slc10a1*, (F) liver *Cyp7b1*, (G) liver *Cyp8b1*, (H) liver *Abcc2*, (I) ileum *Fgf15*, (J) ileum *Fabp6*, (K) ileum *Slc51a*, (L) ileum *Slc51b*, and (M) ileum *Slc10a2*. (N) Normalised protein expression of BSEP and NTCP including quantification relative to Na^+^/K^+^ ATPase. Data are shown as mean ± SD, and individual data points represent individual mice. Liver gene expression is corrected for *Gapdh* and ileum gene expression for *Gapdh* and *Hprt*. Statistical differences were measured using a Kruskal–Wallis one-way ANOVA, ∗*p* ≤0.05. *Abcb11*, ATP binding cassette subfamily B member 11; *Abcc2*, ATP-binding cassette sub-family C member 2; ASBT, apical sodium-dependent bile acid transporter; ASBTi, ASBT inhibitor; BSEP, bile salt export pump; *Cyp7a1*, cholesterol 7 alpha-hydroxylase; *Cyp7b1*, cholesterol 7 beta-hydroxylase; *Cyp8b1*, sterol 12-alpha-hydroxylase; *Fabp6*, fatty acid binding protein 6; *Fgf15*, fibroblast growth factor 15; *Gapdh*, glyceraldehyde 3-phosphate dehydrogenase; *Hprt*, hypoxanthine guanine phosphoribosyltransferase; NTCP, Na^+^-taurocholate cotransporting polypeptide; *Shp*, small heterodimer particle (or *Nr0b2*); *Slc10a1*, solute carrier family 10 member 1; *Slc51a*, solute carrier family 51 subunit alpha; *Slc51b*, solute carrier family 51 subunit beta.

Liver *Cyp7a1* mRNA expression levels were decreased owing to aldafermin treatment, thereby strongly repressing bile salt synthesis in both aldafermin monotherapy and aldafermin + ASBTi combination therapy (Fig. 7A). At the same time, *Shp* signalling was increased in aldafermin + ASBTi (Fig. 7B). Bile salt transporters *Slc51b* and *Abcb11* were downregulated in mice treated with aldafermin + ASBTi compared with placebo and ASBTi monotherapy, whereas *Slc10a1* was increased (Fig. 7C–E). *Cyp7b1* was not affected by treatment, but compared with aldafermin monotherapy, aldafermin + ASBTi combination treatment increased *Cyp8b1* expression (Fig. 7F and G). Aldafermin treatment also did not affect Abcc2 expression (Fig. 7H). In the ileum, aldafermin treatment resulted in the absence of *Fgf15* (Fig. 7I). *Fabp6*, *Slc51a*, and *Slc51b* expression was reduced in the combination treatment group compared with the placebo treatment group (Fig. 7J–L). By contrast, expression of *Slc10a2* was not affected by any treatment (Fig. 7M). Protein expression of BSEP and NTCP was not significantly changed because of treatment, although NTCP expression showed a trend towards higher expression, in line with partial normalisation of intracellular bile salt levels (Fig. 7N).

### Aldafermin + ASBTi strongly reduced faecal bile salt excretion compared with ASBTi monotherapy

**Fig. 8:**
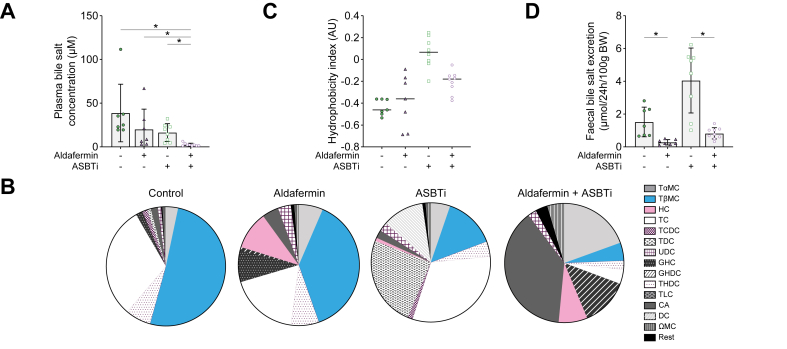
Combination therapy of aldafermin + ASBTi reduced plasma bile salt concentrations and faecal bile salt excretion. (A) Plasma bile salt concentration. (B) Plasma bile salt pool composition. (C) Plasma bile salt hydrophobicity index. (D) Faecal bile salt excretion. Data are shown as mean ± SD, and individual data points represent individual mice. Statistical differences were measured using a Kruskal–Wallis one-way ANOVA, ∗*p* ≤0.05. ASBT, apical sodium-dependent bile acid transporter; ASBTi, ASBT inhibitor; BW, body weight; CA, cholic acid; DC, deoxycholic acid; GHC, glycine-conjugated hyocholic acid; GHDC, glycine-conjugated hyodeoxycholic acid; HC, hyocholic acid; TɑMC, taurine-conjugated α-muricholic acid; TβMC, taurine-conjugated β-muricholic acid; TC, taurine-conjugated cholic acid; TDC, taurine-conjugated deoxycholic acid; TCDC, taurine-conjugated chenodeoxycholic acid; THDC, taurine-conjugated hyodeoxycholic acid; TLC, taurine-conjugated lithocholic acid; UDC, ursodeoxycholic acid; ΩMC, Ω-muricholic acid.

Plasma bile salt levels were decreased because of combination treatment compared with placebo, aldafermin, and ASBTi monotherapy (Fig. 8A). The composition of plasma bile salts was also changed; plasma of mice treated with aldafermin + ASBTi contained large fractions of primary bile acid cholic acid and taurine-conjugated α-muricholic acid, whereas plasma of the monotherapy-treated mice contained more taurine-conjugated cholic acid and taurine-conjugated β-muricholic acid (Fig. 8B). Only in mice treated with aldafermin, both as monotherapy and in combination with ASBTi, a significant fraction of unconjugated hyocholic acid was measured. Hydrophobicity of plasma bile salts was not affected by the change in composition (Fig. 8C). Notably, aldafermin cotreatment with ASBTi lowered faecal bile salt excretion to approximately fourfold compared with ASBTi monotherapy, likely reflecting a strong reduction in the presence of bile salts in the colon of mice treated with aldafermin + ASBTi (Fig. 8D).

## Discussion

Combination therapies that simultaneously target multiple aspects of cholestasis-induced liver injury offer a promising approach for improving liver health. In this study, we investigated the effectiveness of a combination therapy consisting of an intestinal ASBTi along with both FXR-dependent and FXR-independent bile salt repression. Our results showed that combination therapies were more effective than monotherapy in improving liver health in a preclinical cholestatic model. Specifically, combination therapy resulted in reduced liver injury markers, improved liver histology, and decreased inflammatory and fibrotic gene expression in the liver. Importantly, combination therapy also resulted in reduced faecal bile salt excretion compared with ASBTi monotherapy, which may help minimise the risk of side effects, such as bile acid-induced diarrhoea.

Several other monotherapies are currently under investigation for their potential to improve cholestasis-induced liver injury and related symptoms. Most of these monotherapies target hepatic bile acid accumulation or toxicity and are in various levels of clinical development. For instance, we have previously shown that bulevirtide (previously called Myrcludex B), a peptide that inhibits NTCP, can be effective in mouse models of acute cholestasis.^22^ Bulevirtide can further increase circulating bile salt levels, which is in contrast to ASBTi, which results in lower circulating plasma bile salt levels caused by bile salt loss in the faeces. In 2022, Strängberg *et al.*^23^ presented their preclinical work on a dual ASBT/NTCP inhibitor, in which combined inhibition of total body ASBT with hepatic NTCP increased both urinary and faecal bile salt excretion. Earlier, we have shown that inactivation of renal ASBT can result in strongly increased excretion of bile salts in urine and thereby improve cholestasis-induced liver injury.^19^ A systemic ASBTi that also induces urinary bile salt excretion (A3907) increased urinary bile acid elimination, reduced serum bile acid levels, and prevented body weight loss, while improving markers of liver injury in bile duct ligated mice and being safe and well tolerated in healthy volunteers.^24^

FXR agonists such as OCA and cilofexor, as well as aldafermin and ASBTi, are already extensively studied as monotherapy or in combination with UDCA in phase II and III clinical trials for various cholestatic liver diseases.^5^^,^^8^^,^^16^^,^^25^^,^^26^ Furthermore, OCA is licensed by the FDA and EMA for use as a second-line therapy in individuals with PBC, and several ASBTi have recently been added to the list of treatment options for Alagille syndrome and PFIC.^13^^,^^14^^,^^27^

Although some of these individual therapies have shown promising results, they also come with unwanted side effects or lack clinical efficacy. By combining these therapies, the risk of side effects may be reduced^18^ and efficacy increased. For example, ASBTi are associated with bile acid-induced diarrhoea. Inhibiting ASBT also upregulates bile salt synthesis owing to reduced FXR–FGF15 signalling from enterocytes to the liver.^28^ However, when combined with FXR agonists or aldafermin, faecal bile acid excretion may be reduced. In fact, when ASBT-deficient mice are treated with either FGF15 AAV or the FXR agonist GW4046, faecal bile salt excretion was limited.^29^ Our data show that, compared with ASBTi monotherapy, combined treatment of ASBTi with clinically applied FXR agonists or aldafermin lowered faecal bile acid excretion. We hypothesise that reducing the high bile salt load in the colon might lower gastrointestinal symptoms; however, preclinical mouse models are not sufficient to test this hypothesis.

Furthermore, OCA monotherapy is associated with a dose-dependent increase in pruritus,^30^ and strong repressed bile salt synthesis with aldafermin can lead to hypercholesterolaemia.^11^^,^^31^ In contrast, intestinal ASBT inhibition has been shown to reduce pruritus and increase faecal lipid excretion in line with its hepatoprotective effects in a mouse model of non-alcoholic fatty liver disease.^32^ This suggests that individual treatments can complement each other when used as a combination therapy, including both lipid profile and faecal bile salt excretion. For two of the tested combinations with ASBTi, that is, cilofexor and aldafermin, combination treatments potentially allow for lower dosages of the individual therapies, which may lower the risk of side effects. Although it is not known for cilofexor, inhibition of ASBT likely lowers ileal uptake of conjugated OCA, here reflected in dampened effects of OCA with respect to *Cyp7a1* repression. Higher dosing is not a straightforward solution to compensate for this, as we observed serious intestinal side effects at 30 mg/kg dosing.

With the exception of cilofexor monotherapy, combination therapies seem more effective in lowering plasma liver enzymes ALT and AST. For aldafermin-treated mice, plasma FGF19 concentrations of ∼600 pg/ml were measured, whereas persons with extrahepatic cholestasis reach levels higher than 2 ng/ml.^33^ This implies that the dosing concentration is modest. For OCA and cilofexor, the dosages exceed those in current clinical use, and this may contribute to why an additive effect of the combination of cilofexor with ASBTi on plasma liver enzymes is less prominent.

The primary outcome regularly used in cholestasis-related research in adults is ALP, of which a reduction equals improvement of disease. In our study, monotherapy with OCA or cilofexor and combination therapy cilofexor + ASBTi had no effect on plasma ALP. A phase II clinical trial in people with PSC described a similar finding, where after 12 weeks of aldafermin treatment, no improvement in ALP was found, despite improvements in Pro-C3, ALT, and AST.^25^ Similar findings were obtained in a phase III trial in patients with PSC,^9^ suggesting that ALP as the main primary outcome parameter may miss some elements of cholestatic injury. As ALP is mainly bone-derived in children, this marker is mostly replaced by serum bile salt levels and pruritus scores as primary outcomes in trials for paediatric cholestatic diseases. In this study, liver enzymes ALT and AST, as well as plasma bile salts levels, are most strongly reduced in the combination groups.

A striking finding is that even though OCA and cilofexor are both FXR agonists, the effect of combining them with ASBTi on plasma bile salt composition was qualitatively different. Cilofexor combination therapy with ASBTi resulted in more hydrophilic circulating bile salts, which are less toxic. Although plasma bile salt concentration and composition are determined by a large number of parameters, including synthesis, intestinal transit, and hepatic and microbial metabolic conversion, one likely explanation is the difference in hepatic FXR agonism between OCA + ASBTi and cilofexor + ASBTi combination therapies. Only the latter combination resulted in strong hepatic induction of *Shp* expression, and *Cyp7a1* and *Cyp8b1* repression. This suggests that OCA-mediated FXR agonism is mostly intestine restricted when combined with ASBTi, whereas cilofexor is able to activate also hepatic FXR.

Persons with PSC treated with aldafermin had lower plasma secondary bile acids, whereas a reduction in primary bile acid glycocholic acid was found in aldafermin-treated patients with PBC.^25^^,^^26^ Inhibiting intestinal ASBT lowers primary bile salts in mice and humans, creating a more hydrophobic bile salt pool.^34^^,^^35^ We see a similar trend, but the combination of ASBTi with aldafermin or cilofexor lowered or normalised the hydrophobicity and therefore seems relevant in the context of cholestasis.

### Conclusions

Taken together, our results highlight the potential of combination therapies to improve liver health and reduce the risk of side effects in cholestatic liver diseases. In preclinical cholestatic models, combining repressed bile salt synthesis with pharmacological blockade of intestinal bile salt re-uptake reduces plasma liver injury markers, improves liver histology by lowering inflammatory and fibrotic markers, and reduces faecal bile salt excretion levels. Further (clinical) studies are required to investigate whether the indicated combination therapies reduce the risk of developing unwanted side effects including pruritus and diarrhoea in patients with cholestasis.

## Financial support

This work was supported by grants from the Netherlands Organization for Scientific Research (Vidi 91713319 and Vici 09150182010007 to SFJVDG).

## Authors’ contributions

Contributed to the design of the experiments: RFK, BAN, UB, RPJOE, SFJVDG. Performed experiments: RFK, IB, RDJVD. Performed drafting and initial review of the manuscript: RFK, IB, UB, RPJOE, SFJVDG. Were involved in analysis and interpretation of data and have read and approved the final manuscript: all authors.

## Data availability statement

The data that support the findings in this study are included in the manuscript and/or its Supplementary figures.

## Conflicts of interest

The authors declare no conflict of interest with regard to this work.

Please refer to the accompanying ICMJE disclosure forms for further details.
