# Supplementary material for: Combined inhibition of bile salt synthesis and intestinal uptake reduces cholestatic liver damage and colonic bile salts in mice
Source: JHEP Rep. 2023 Sep 25;6(1):100917. doi: 10.1016/j.jhepr.2023.100917 (PMC10701132; doi:10.1016/j.jhepr.2023.100917)
Supplement: Multimedia component 1 [file mmc1.pdf]

# Combined inhibition of bile salt synthesis and intestinal uptake reduces cholestatic liver damage and colonic bile salts in mice

Roni F. Kunst, Isabelle Bolt, Rychon D. J. van Dasselaar, Bart A. Nijmeijer, Ulrich Beuers, Ronald P.J. Oude Elferink, Stan F.J. van de Graaf

Table of contents

Supplementary Materials and Methods……………………………………………………2

Fig. S1……………………………………………………………………………………6

Fig. S2……………………………………………………………………………………7

Fig. S3……………………………………………………………………………………8

Supplementary References……………………………………………………………9

**Supplementary Materials and Methods**

*Animals and experimental design*

The first cohort of mice receiving OCA were dosed with 30mg/kg/day via oral gavage. Mice did not tolerate this dose of OCA resulting in body weight loss requiring sacrifice of 6 (out of 12) mice within 2 days. For the remainder of the experiment (day 2 – end), mice received 10 mg/kg/day OCA. All mice from the second cohort received 10mg/kg/day from start of the experiment (Supplementary Methods Fig. 1).

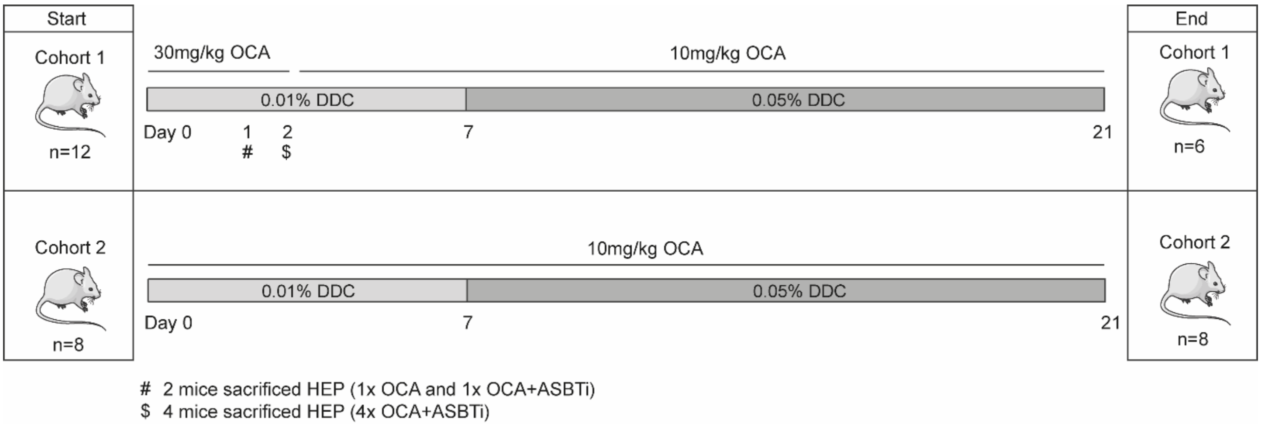


**Supplementary Methods Fig. 1: Detailed description of the OCA dosing strategy in mice, divided in two different cohorts.**

*Adeno associated virus (AAV) production*

Human embryonic kidney (HEK) 293T cells were plated in 20cm culture dishes and co-transfected with helper plasmid pDP8 (containing REP gene from AAV2, cap gene from AAV8 and the adenovirus helper genes E2A, E4 and VA-RNA) and pAAV_mPGK_NGM282[1] or a control GFP containing expression vector in a pTRCGW[2] backbone with polyethylenimine (PEI). 66hours after transfection, cells were harvested and AAV was purified using an Iodixanol (OptiPrep, 07820) gradient and Ultracell 100K centrifugal filterunits (Amicon, UFC910024). Titer determination was performed on inactivated purified AAV by RT-qPCR as described before[3].

*RNA isolation, cDNA synthesis and RT-qPCR*

Gene expression was measured by RT-qPCR as described before[3]. Mice were excluded when gene expression was >2SD aberrant in 3 or more genes. Primers used for RT-qPCR are shown in Supplementary Methods Table 1.

**Supplementary Methods table 1: RT-qPCR primers for gene expression**

| Gene | FW (5→3) | RV (5→3) | Size (bp) |
|---|---|---|---|
| *m_Hprt* | CCTAAGATGAGCGCAAGTTGAA | CCACAGGACTAGAACACCTGCTAA | 86 |
| *m_Gapdh* | GACAACTCATCAAGATTGTCAGCA | TTCATGAGCCCTTCCACAATG | 106 |
| *m_Slc10a1* | TGGCTACCTCCTCCCTGATG | GCCAGGTTGTGTAGGAGGAT | 380 |
| *m_Slc10a2* | GGGGTATCTTCGTGGGCTTC | TGCTAACACTGAGGTCCATGTC | 189 |
| *m_Abcb11* | TGGAAAGGAATGGTGATGGG | CAGAAGGCCAGTGCATAACAGA | 77 |
| *m_Slc51a* | GGCATCTATGACCCAGGAGA | TGGATCCCATGTTCTGTTCA | 151 |
| *m_Slc51b* | GACCACAGTGCAGAGAAAGC | ATTCCAAGGAGCCGCATCT | 102 |
| *m_Tnfa* | CATCTTCTCAAAATTCGAGTGACAA | TGGGAGTAGACAAGGTACAACCC | 175 |
| *m_Col1a1* | CCCTGGTCCCTCTGGAAATG | GGACCTTTGCCCCCTTCTTT | 72 |
| *m_Mcp-1* | CTTCTGGGCCTGCTGTTCA | CCAGCCTACTCATTGGGATCA | 127 |
| *m_aSma* | ACTACTGCCGAGCGTGAGAT | AAGGTAGACAGCGAAGCCAG | 452 |
| *m_Shp* | GGCACGATCCTCTTCAACCC | GGCACCAGACTCCATTCCAC | 287 |
| *m_Fabp6* | GAGACGTGATTGAAAGGGGA | TTACGCGCTCATAGGTCACA | 294 |
| *m_Cyp7a1* | CTGGGGGATTGCTGTGGTAG | CTGTGTCCAAATGCCTTCGC | 315 |
| *m_Timp1* | TGGGGAACCCATGAATTTAG | ATCTGGCATCCTCTTGTTGC | 127 |
| *m_Cyp8b1* | TGTCTACTCCCTACTGGGGC | GAGAGCCACCTTATCTCCGC | 505 |
| *m_Cyp7b1* | AGCTTGGTCTGCCTGGAAAG | TGAGTGGAGGAAAGAGGGCT | 157 |
| *m_Abcc2* | TCCAGGACCAAGAGATTTGC | TCTGTGAGTGCAAGAGACAGG | 107 |

*Bile salt analysis*

Bile salt concentrations in blood plasma and feces were measured by reverse phase High Performance Liquid Chromatography (HPLC) as described before[4]. One mouse was excluded as plasma bile salt levels were >10 times increased compared to group average, which could not be explained and was therefore considered an outlier (Supplementary Methods Fig. 2).

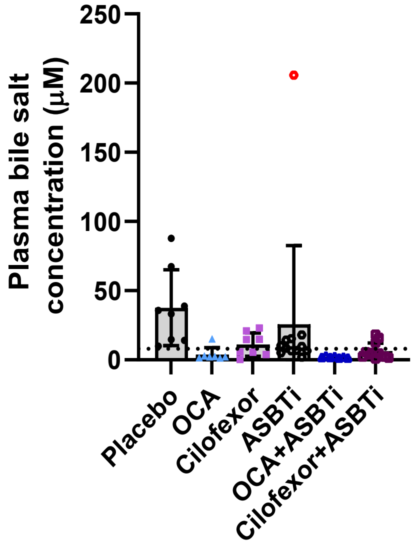


**Supplementary Methods Fig. 2: Plasma bile salt concentrations including an outlier (in red).**

*Histology and immunohistochemistry*
Formalin fixed and paraffin-embedded mouse liver tissues were processed and stained for Hematoxylin and Eosin (H&E) and anti- cytokeratin 7 (CK7) as described before[3]. Sirius Red stain was performed by dissolving Direct Red 80 (Sigma, Houten, The Netherlands) in a 1.3% saturated aqueous solution of picric acid (Sigma). Digital imaging of the sections was done with an Olympus BX-51 microscope, equipped with a 200x objective and was scored blinded by three researchers. Representative images are shown. Anti- DESMIN (1:100, D93F5, Cell Signaling, #5332), collagen 1A1 (COL1A1, 1:200, E8F4L, Cell Signaling #72026), alpha smooth muscle actin (αSMA, 1:500, D4K9N, Cell Signaling #19245), and platelet-derived growth factor receptor beta (PDGFRβ, 1:100, 28E1, Cell Signaling #3169) immunohistochemistry stainings were performed on 8μm cryo-sections made with the Leica CM1950 cryostat. Sections were incubated with BLOXALL blocking solution (Biozol), before o/n incubation at 4ºC with primary rabbit anti mouse antibody. A secondary alkaline phosphatase-conjugated polyclonal goat anti-rabbit (IgG, PA5-51057, Immunologic) antibody was used to visualize positive staining with SignalStain Vibrant Red Alkaline Phosphatase Substrate Kit (Cell Signaling #76713). Additionally, hematoxylin (Mayer's, SLCN6531) counterstain was performed. Liver damage was scored based on histological staining, independently and blinded by 2-3 researchers.

*Plasma biochemistry*
Plasma biomarkers for liver injury and cholestasis, ALT, AST and alkaline phosphatase diluted in 0.9% NaCl and measured by routine clinical biochemistry testing at the LAKC, Amsterdam Medical Center. These biomarkers are measured by photometric assay tests on the Roche Cobas c502/702 analyzer (Roche Diagnostics).

*Immunoblotting*
200mg mouse liver was homogenized for 30min in 1 mM $NaHCO_3$ in the Qiagen TissueLyser LT using stainless steel beads (Qiagen 69989). Subsequently, protein lysates were incubated on ice for 15min before a glass douncer was used to further homogenize the lysate. After centrifugation to remove cell debris, the supernatant was transferred and used to separate membrane protein from cytosolic protein using the Beckman Coulter Optima XE-90 ultracentrifuge, 43000rpm, 4C. Protein quantity was determined by the Pierce BCA protein assay (Thermo Fisher, 23225) and 30μg membrane protein was used for Semi-Dry Western Blot. Protein was separated on a 4% to 15% polyacrylamide gradient gel and transferred to a polyvenylidene fluoride (PVDF) membrane using the Trans-blot Turbo transfer system (Biorad). To reduce nonspecific antibody binding, the membrane was incubated for 1h at room temperature with 5% milk in 1x TBS-Tween20. Antibodies against NTCP (rabbit K4, 1:1000, gift from Bruno Stieger), BSEP (rabbit K12, 1:1000, gift from Bruno Stieger), B-actin (AC-15, mouse IgG1, 1:4000, Sigma A5441) and ATP1A1 (C356-M09, 1:1000, gift from J.B. Koenderink) were diluted in 1% milk in 1x TBS-Tween20 and incubated overnight at 4ºC. A 1hr incubation at RT with secondary antibody goat-a-

rabbit (IgG, 1:4000, BioRad 170-6515) or goat-a-mouse (IgG, 1:4000, BioRad 170-6516) conjugated with horseradish peroxidase was used to visualize protein expression using ECL substrate and $H_2O_2$ on the Chemidoc MP (Biorad). Non-processed western blots are shown in Fig. S3.

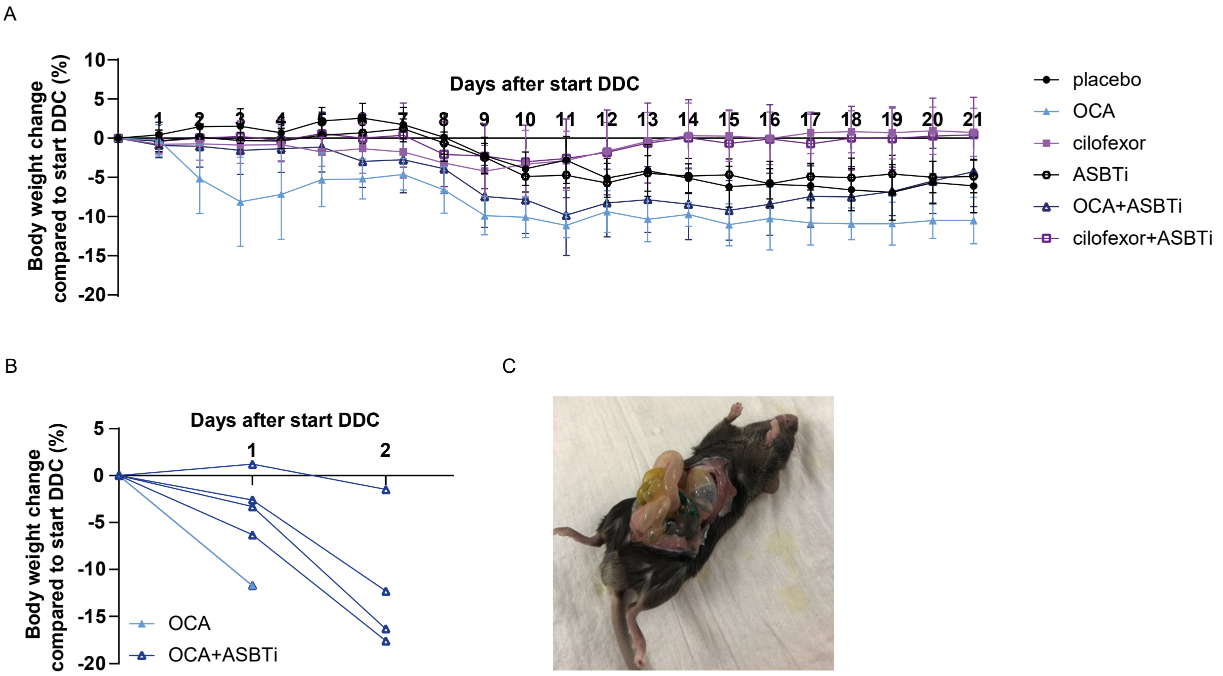


**Fig. S1. 30mg/kg/day OCA dosing was not well-tolerated by male wild type C57BL/6JOlaHSD mice.** (A) Bodyweight change over time, without OCA treated mice that died within the first two days of treatment (B) Drop-out of 30mg/kg/day OCA treatment (C) Post-mortem section of a mouse receiving 30mg/kg OCA before reaching human endpoint. Data is shown as mean±standard deviation

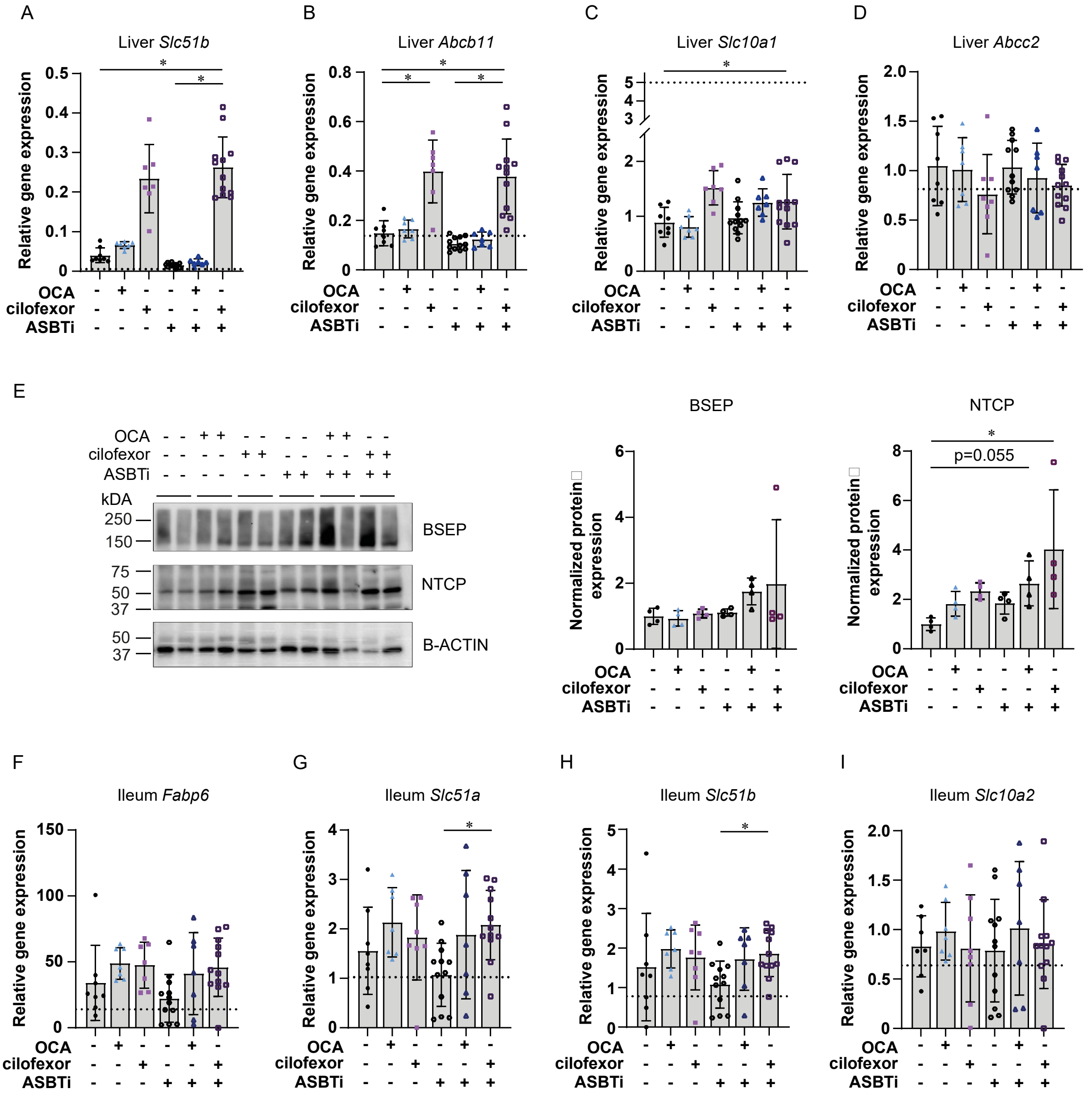


**Fig. S2. Cilofexor induced bile salt signaling, more than OCA.** Gene expression of (A) liver Slc51b (B) liver Abcb11 (C) liver Slc10a1 (D) liver Abcc2 (E) Normalized protein expression of BSEP and NTCP including quantification relative to B-ACTIN. Representative blots. (F) ileum Fabp6 (G) ileum Slc51a (H) ileum Slc51b and (I) ileum Slc10a2. Data is shown as mean±standard deviation, individual data points represent individual mice. Gene expression is relative to Gapdh (liver) and Hprt (ileum). Healthy controls (n=3) are indicated by the dotted line. Statistical differences were measured with a Kruskal Wallis One-Way ANOVA test, *p≤0.05

A

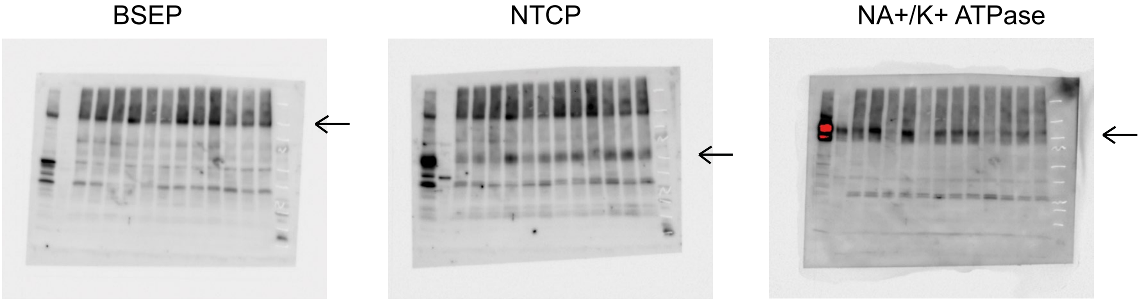


| | |
|---|---|
| healthy control - No DDC | lane 1 |
| negative control | lane 2 |
| placebo | lane 3,4,5 |
| aldafermin | lane 6,7,8 |
| ASBTi | lane 9,10,11 |
| aldafermin+ASBTi | lane 12,13,14 |

B

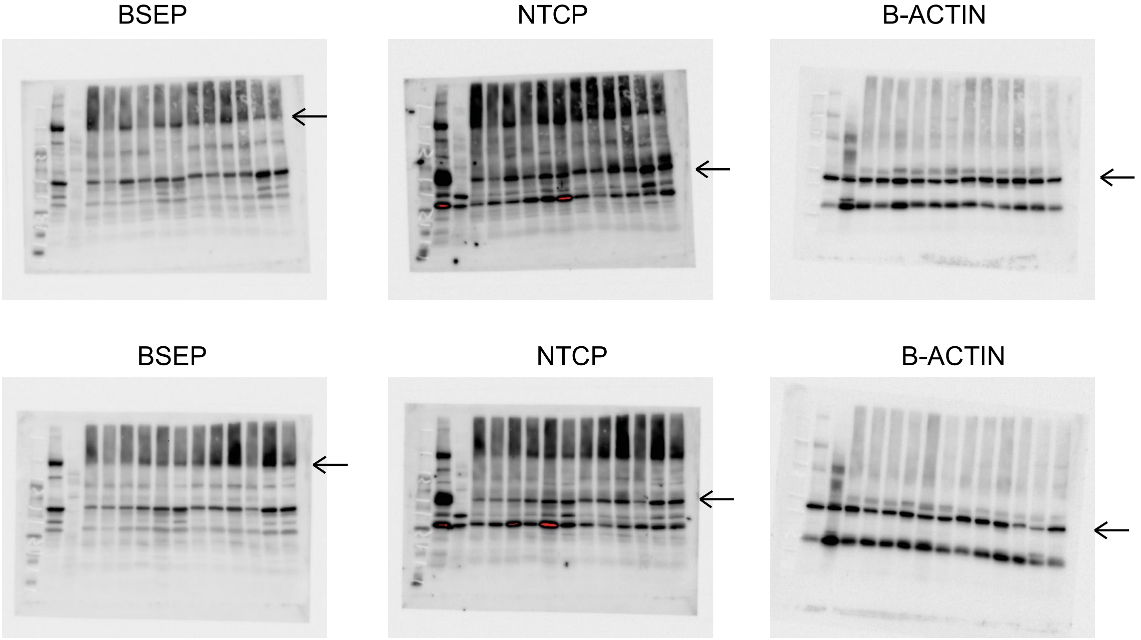


| | |
|---|---|
| healthy control - No DDC | lane 1 |
| negative control | lane 2 |
| placebo | lane 3,4 |
| OCA | lane 5,6 |
| cilofexor | lane 7,8 |
| ASBTi | lane 9,10 |
| OCA+ASBTi | lane 11,12 |
| cilofexor+ASBTi | lane 13,14 |

**Fig. S3. Non-processed Western Blots**. A) Aldafermin study, n=3 per condition, BSEP, NTCP and Na+/K+ATPase. B) Cilofexor study, n=4 per condition, BSEP, NTCP and B-ACTIN

## Supplementary References


1. Luo J, Ko B, Elliott M, et al. A nontumorigenic variant of FGF19 treats cholestatic liver diseases. *Sci Transl Med* 2014;6(247):247ra100. doi: 10.1126/scitranslmed.3009098
2. **Ross CJ**, **Twisk J**, **Meulenberg JM**, et al. Long-term correction of murine lipoprotein lipase deficiency with AAV1-mediated gene transfer of the naturally occurring LPL(S447X) beneficial mutation. *Hum Gene Ther* 2004;15(9):906-19. doi: 10.1089/hum.2004.15.906
3. Kunst RF, de Waart DR, Wolters F, et al. Systemic ASBT inactivation protects against liver damage in obstructive cholestasis in mice. *JHEP Rep* 2022;4(11):100573. doi: 10.1016/j.jhepr.2022.100573 [published Online First: 20220827]
4. Slijepcevic D, Kaufman C, Wichers CG, et al. Impaired uptake of conjugated bile acids and hepatitis b virus pres1-binding in na+-taurocholate cotransporting polypeptide knockout mice. *Hepatology* 2015;62(1):207-19.
