# Supplementary material for: Combined inhibition of bile salt synthesis and intestinal uptake reduces cholestatic liver damage and colonic bile salts in mice
Source: JHEP Rep. 2023 Sep 25;6(1):100917. doi: 10.1016/j.jhepr.2023.100917 (PMC10701132; doi:10.1016/j.jhepr.2023.100917)
Supplement: Multimedia component 2 — : [file mmc2.docx]

**Journal of Hepatology**

**CTAT methods**

Tables for a “Complete, Transparent, Accurate and Timely account” (CTAT) are now mandatory for all revised submissions. The aim is to enhance the reproducibility of methods.

- Only include the parts relevant to your study
- Refer to the CTAT in the main text as ‘Supplementary CTAT Table’
- Do not add subheadings
- Add as many rows as needed to include all information
- Only include one item per row

**If the CTAT form is not relevant to your study, please outline the reasons why:**

|  |
| --- |

- 1. **Antibodies**

| **Name** | **Citation** | **Supplier** | **Cat no.** | **Clone no.** |
| --- | --- | --- | --- | --- |
| **Anti-CK7** |  | **Abcam** | **ab181598** | **EPR17078** |
| **Anti-PDGFRb** |  | **Cell Signaling** | **#3169** | **28E1** |
| **Anti-αSMA** |  | **Cell Signaling** | **#19245** | **D4K9N** |
| **Anti-COL1A1** |  | **Cell Signaling** | **#72026** | **E8F4L** |
| **Anti-DESMINE** |  | **Cell Signaling** | **#5332** | **D93F5** |
| **Anti-BSEP** |  | **B. Stieger** |  | **K12** |
| **Anti-NTCP** |  | **B. Stieger** |  | **K4** |
| **Anti-ATP1A1** |  | **J.B. Koenderink** |  | **C356-M09** |
| **Anti-βACTIN** |  | **Sigma Aldrich** | **#5441** | **AC-15** |

- 1. **Cell lines**

| **Name** | **Citation** | **Supplier** | **Cat no.** | **Passage no.** | **Authentication test method** |
| --- | --- | --- | --- | --- | --- |
|  |  |  |  |  |  |

- 1. **Organisms**

| **Name** | **Citation** | **Supplier** | **Strain** | **Sex** | **Age** | **Overall n number** |
| --- | --- | --- | --- | --- | --- | --- |
| **Wild type mice** |  | **Envigo** | **C57BL/6JOlaHsd** | **M** | **>8w** | **94** |

- 1. **Sequence based reagents**

| **Name** | **Sequence** | **Supplier** |
| --- | --- | --- |
|  |  |  |

- 1. **Biological samples**

| **Description** | **Source** | **Identifier** |
| --- | --- | --- |
|  |  |  |

- 1. **Deposited data**

| **Name of repository** | **Identifier** | **Link** |
| --- | --- | --- |
|  |  |  |

- 1. **Software**

| **Software name** | **Manufacturer** | **Version** |
| --- | --- | --- |
| **LinRegPCR** | **Ramakers et al. (2003) Neuroscience letters.** | **12.5** |
| **GraphPad** | **PRISM** | **9** |
| **CellSens Entry** | **Olympus** | **4.1** |

- 1. **Other (e.g. drugs, proteins, vectors etc.)**

| **ASBT inhibitor** | **Linerixibat** | **GSK2330672** |
| --- | --- | --- |
| **Obeticholic acid** | **Cayman Chemicals** | **11031** |
| **Cilofexor** | **GS-9674** | **MedChemExpress** |
| **pAAV_NGM282** | **See manuscript** | **See supplementary methods** |
| **pTRCGW** | **See manuscript** | **See supplementary methods** |

- 1. **Please provide the details of the corresponding methods author for the manuscript:**

| **Stan van de Graaf, Ph.D.,**  **Tytgat Institute for Liver and Intestinal Research, Academic Medical Center, Meibergdreef 69-71, 1105 BK Amsterdam, The Netherlands.**  **E-mail: k.f.vandegraaf@amsterdamumc.nl**  **Tel: +31-020-5668832**  **Fax: +31-020-5669190** |
| --- |

**2.0 Please confirm for randomised controlled trials all versions of the clinical protocol are included in the submission. These will be published online as supplementary information.**

|  |
| --- |
